# Estimation of Cancellous Changes Using Fractal Analysis in Patients with Periodontitis

**DOI:** 10.3390/biomedicines11092547

**Published:** 2023-09-16

**Authors:** Sukanya Mishra, Manoj Kumar, Lora Mishra, Swagatika Panda, Saurav Panda, Natalia Lewkowicz, Barbara Lapinska

**Affiliations:** 1 Department of Periodontics and Oral Implantology, Institute of Dental Sciences and SUM Hospital, Siksha ‘O’ Anusandhan University, Bhubaneswar 751003, India; drmishrasukanya@gmail.com (S.M.); sauravpanda@soa.ac.in (S.P.); 2 Department of Conservative Dentistry and Endodontics, Institute of Dental Sciences and SUM Hospital, Siksha ‘O’ Anusandhan University, Bhubaneswar 751003, India; loramishra@soa.ac.in; 3 Department of Oral Pathology and Microbiology, Institute of Dental Sciences and SUM Hospital, Siksha ‘O’ Anusandhan University, Bhubaneswar 751003, India; swagatikapanda@soa.ac.in; 4 Department of Periodontology and Oral Diseases, Medical University of Lodz, 251 Pomorska St, 92-213 Lodz, Poland; natalia.lewkowicz@umed.lodz.pl; 5 Department of General Dentistry, Medical University of Lodz, 251 Pomorska St, 92-213 Lodz, Poland

**Keywords:** dentistry, periodontology, periodontitis, fractal dimension, cancellous bone

## Abstract

Periodontal disease is a broad term given when the periodontium is affected by inflammation. There are several methods to diagnose periodontitis, but no method to quantify the cancellous bone is presently used. For this purpose, a non-invasive tool that is efficient in analyzing bone quality called fractal analysis can be employed. The objective of the study was to utilize fractal dimension (FD) to evaluate cancellous patterns of interproximal alveolar bone using a digital intraoral periapical radiograph (IOPAR) in different clinical presentations of periodontitis classified according to the latest classification by the World Workshop for Periodontal and Peri-Implant Diseases and Conditions, 2017 (WWDC, 2017). The study aimed to numerically calculate the periodontitis changes in the cancellous bone around the affected tooth on an IOPAR using fractal analysis and to provide an additional criterion for the staging of periodontitis. In this cross-sectional observational study, 75 patients were selected and equally allotted to five groups based on the staging system proposed by the WWDC, 2017. The region of interest was selected on the IOPA radiograph of the tooth with the site having the most significant clinical attachment loss, and FD was calculated using Image J analysis. The association of gender and age with stages of periodontitis was studied using the chi-square test of independence. A comparison of % bone loss and fractal dimension among stages was studied by using the non-parametric Kruskal Wallis test. The relationship between % bone loss and fractal dimension within stages and gender was studied by using the Karl Pearson correlation. A receiver operating characteristic (ROC) curve analysis was performed for FD value as a marker of periodontitis patients. We demonstrated that the FD values decreased significantly with the increase in stages (*p* < 0.0001). The overall relationship between % bone loss and FD showed a significantly strong negative correlation of −0.739 (*p* < 0.0001), except for stages III and IV. FD can be utilized as a quantitative method for detecting cancellous bone changes in different stages of periodontitis, aiding in diagnosing periodontitis.

## 1. Introduction

Periodontal disease is a comprehensive term when the periodontium, which comprises a set of structures that support the teeth, is affected by inflammation. Periodontitis is characterized by proinflammatory cascades, dysbiosis of oral microbiota, and involvement of cells and mediators from innate and adaptive immunity [1].

Clinical and radiographic assessments of periodontal tissue are requisites for diagnosing periodontal disease. In a comprehensive clinical exam, around each tooth, at four or six sites, the following parameters are measured: presence of dental plaque [2], pocket probing depths (PD) [3], bleeding on probing (BOP) [4], clinical attachment level (CAL) [5], furcation involvement [6], suppuration/exudate mobility [7] and occlusal trauma [8]. The recent periodontitis classification given in 2017 by the AAP and the European Federation of Periodontology (EFP) at the World Workshop for Periodontal and Peri-Implant Diseases and Conditions (WWDC) [9] measures the CAL and, along with the severity, it includes few primary criteria for grading of periodontitis. It confides radiographic bone loss or attachment loss over the span of 5 years as direct evidence and case phenotype and percentage of bone loss as indirect evidence. It also includes some secondary criteria, such as smoking and diabetes as risk factors, inflammatory burden, and biomarker indicators of bone loss. Other diagnostic methods for periodontal disease include commercial assays that use the inflammatory exudates for biomarker tests, which are not widely used because of their low predictive values and cost, and image analysis of dental radiographs or three-dimensional scans. However, in intraoral periapical radiographs, changes in the bone are visible when nearly 30–50% of the bone mineral resorbs [10]; thus, early stages of periodontitis might be overlooked, especially by a non-periodontist. For early diagnosis of periodontal disease, to improve prognosis and treatment, a detailed quantification of the alveolar bone is crucial.

The present world has welcomed digitization, thus eradicating manual errors, improving the visualization of the images, and helping to extract data from the images, such as the alveolar bone’s crest height, bone texture, and bone mass. Bone mass measurement is based on a strong correlation between the bone mass of the alveolar bone and variations in the digital greyscale value [11]. Enhanced processing and strict standardization of projection geometry radiographs have improved the quality of current radiographs to allow for more sensitive and accurate measurements. This can reduce both intra-rater and inter-rater variability. The significance of radiographic image processing lies in gathering information in a non-invasive manner [12].

Fractal dimension (FD) analysis is one such non-invasive method. The word “fractal” is attained from the Latin word “fractus” which means “fracture” [13]. It can be described as the geometry of self-similar forms, and its applicability lies in characterizing complex self-similar shapes in a quantitative manner [14]. Therefore, the concept of fractal dimension quantifies the complexity of a structure by providing a quantitative parameter. It characterizes self-similarity, defined as when a part of an object, after intervention (scaling) by an arbitrary factor, appears the same as the whole object [15]. White and Rudolph [16] processed the digital medical images and then, in a computer program (ImageJ software, https://imagej.nih.gov/ij/), quantified the morphologic features of cancellous bone using the box-counting method. Their paper described the standardized stages that helped highlight the cancellous bone in the digital images. Earlier studies in this context aimed to determine if cancellous bones are fractal. The results have shown that they have an indeterminate perimeter, characteristic of theoretical fractal shapes [17].

Studies have been conducted to analyze the fractal properties of cancellous bone affected with periodontitis [18,19], as well as around dental implants [20,21]. However, the studies have used the earlier 1999 classification of periodontitis by the American Academy of Periodontology (AAP), which had its limitations. The current classification of periodontitis, released by the AAP and the European Federation of Periodontology (EFP) at the World Workshop for Periodontal and Peri-Implant Diseases and Conditions (WWDC) in 2017 [9], employs radiographic changes for both staging and grading of periodontitis. In fact, it was suggested that analysis of bone loss may help screening for periodontitis, especially by non-periodontists; therefore, further facilitation in analysis of radiographs by a clinician is welcomed.

The aim of the study was to estimate changes caused by periodontitis in the cancellous bone around the affected tooth on a periapical radiograph using FD analysis with the following objectives:

1. To determine the relationship between the fractal dimension of the cancellous bone and different stages of periodontal disease.
2. To estimate the changes caused by periodontitis.
3. To provide an additional criterion for the staging of periodontitis.

## 2. Materials and Methods

The study was a cross-sectional observational study conducted among the patients visiting the Department of Periodontics and Oral Implantology, Institute of Dental Sciences and SUM Hospital, SOA (Deemed to be University), Bhubaneswar. The study was carried out in accordance with the ethical guidelines of the Helsinki Declaration. The ethical committee of the Institute of Medical Sciences and SUM Hospital, SOA University approved the study with IEC no: ECR/627/Inst/OR/2014/RR/20. The power set of the study was 80%, and the calculated sample size according to the FD values of critical articles was 15 per group [18,22,23].

In this study, mainly the association of age and gender with groups has been studied by the chi-square test of independence. Therefore, the sample size determination has been performed for the chi-square test of independence.

χ^2^ tests-Goodness-of-fit tests: Contingency tables

Analysis: A priori: Compute required sample size

Input: Effect size w = 0.5

α err prob = 0.05

Power (1-β err prob) = 0.80

Df = 12

Output: Noncentrality parameter λ = 17.5000000

Critical χ^2^ = 21.0260698

Total sample size = 70

Actual power = 0.8046698

The sample size for this study is 75 @ 15 per group, which is higher than the minimum sample size of 70 to achieve a power of the test of 0.80 for a 0.05 level of significance.

### 2.1. Patients’ Selection

Inclusion and exclusion criteria are presented in Table 1.

For the study, 75 patients were selected. Out of the 75 patients, 15 were allotted to the healthy control group, and the rest were allotted to four groups (Table 2) based on the staging system proposed by the Classification of Periodontal and Peri-Implant Diseases and Conditions, 2017—stage I, II, III, and IV. The clinical parameters, i.e., PD and CAL, were recorded, and the diagnosis of periodontitis was established based on the 2017 Classification. Subjects with PD ≤ 3 mm with full mouth BOP < 10%, without any history or evidence of active periodontitis in the past, were considered as the healthy controls. The study procedure was explained to all the participants, and written informed consent was taken before the study.

All clinical periodontal measurements were performed by a single calibrated examiner SM. A periodontal probe (UNC 15 Hu-Friedy, Chicago, USA) was introduced into the gingival sulcus, parallel to the long axis of the tooth. The distance from the gingival margin to the bottom of the clinical pocket was measured at four sites (mesiobuccal, distobuccal, mesiolingual, and distolingual) of all the teeth present. Similarly, CAL was measured interdentally from the CEJ to the base of the pocket.

A digital intraoral periapical radiograph (IOPAR) of the site of greatest clinical attachment loss was taken by a single calibrated examiner SM, and digitization was performed. IOPAR images were obtained using a periapical radiography device with standard #2-size CMOS Sensor and Sopro imaging software (Version 2.41, developed by COMEG, La Ciotat, France). The IOPARs were taken with parallel technique at 70 kVp, 7 mA, and 0.2 sn exposure time. The percentage of radiographic bone loss was calculated using the absolute method in the tooth with the maximum amount of bone loss using an open-source image processing software program (ImageJ, National Institutes of Health, Bethesda, MD, USA) [24].

% of bone loss = Distance from CEJ to defect/Distance from CEJ to apex (length of the root)(1)

After calculation of percentage of radiographic bone loss, the tooth with the maximum amount of bone loss was considered for further analysis.

A rectangular region of interest (ROI) was selected for each subject, depending on the amount of bone loss. Further processing of the ROI was performed, and the box-counting algorithm of the image J software was used to calculate the fractal dimension (FD).

**Figure 1:**
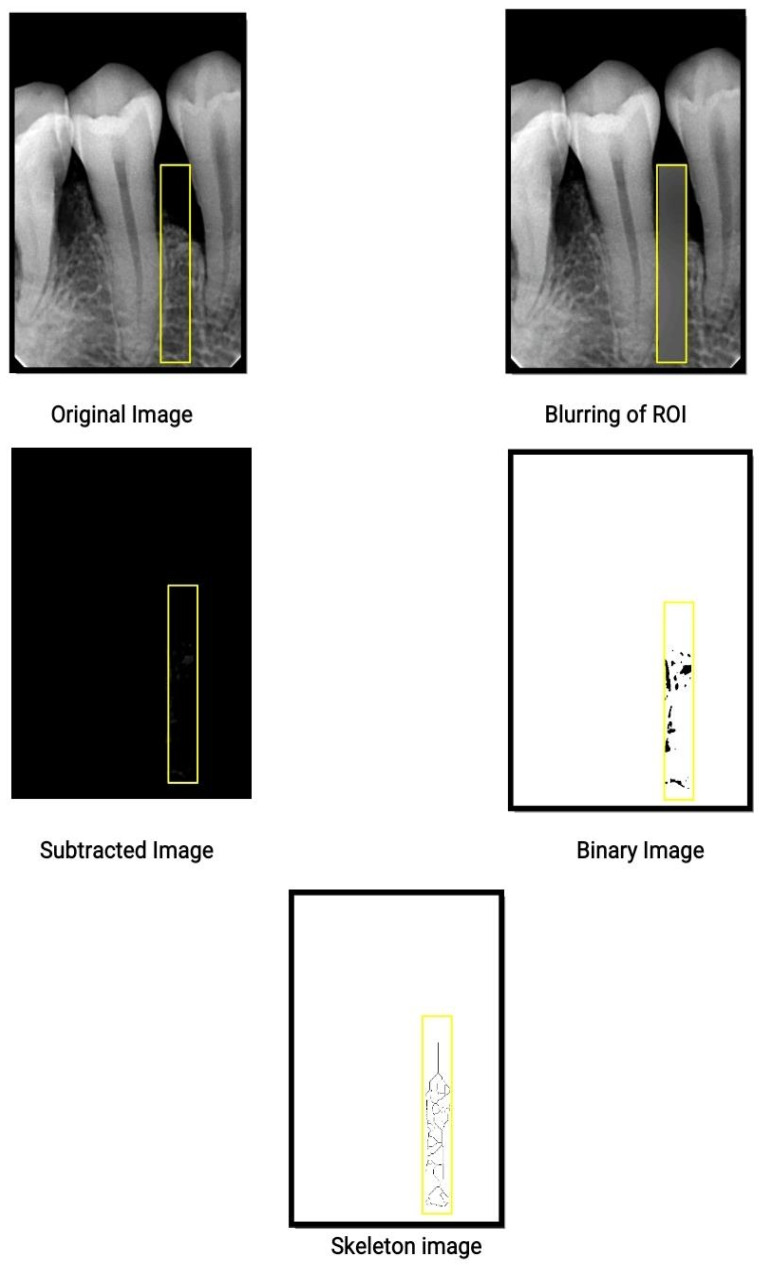
Sample steps for radiographic analysis. Outlined yellow box correspond to the selected ROI.

The rectangular area from the CEJ to the apex of the root in the interproximal region was chosen for every subject as the ROI. Even when the ROI included a part of the root or periodontal ligament (PDL), the analysis excluded these fragments. The reason behind this is that bony trabeculae are dendritic in nature and the skeletonization step of the image process removes everything that is not dendritic. The ROI areas were cropped and drawn using White and Rudolph’s method [16] of image processing using Image J software. FD was calculated using the box-counting algorithm in the software. The sequence followed was blurring of the image using a Gaussian filter, subtraction of the blurred image from the original image, and binarization and skeletonization, as shown in Figure 1.

### 2.2. Statistics

The data collected in the study were scrutinized, codified, and entered into the IBM SPSS Statistics, 24.0 software, www.spss.co.in assessed on 01 July 2022, for analysis. Categorical variables, such as age group and gender, were determined by using a frequency distribution procedure. Association of age group and gender with stages was determined using the cross-tabulation procedure, and their association was studied using the chi-square test of independence. Comparisons of % bone loss and fractal dimension among stages were studied by using the non-parametric Kruskal–Wallis test. The normality of the test was determined using the Shapiro–Wilk test. The relationship between % bone loss and fractal dimension within stages and gender was determined by using the Karl Pearson correlation. A receiver operating characteristic (ROC) curve analysis was performed for the FD value as a marker of periodontitis patients; *p* < 0.05 was considered to indicate a cut-off for statistical significance.

## 3. Results

**Figure 2:**
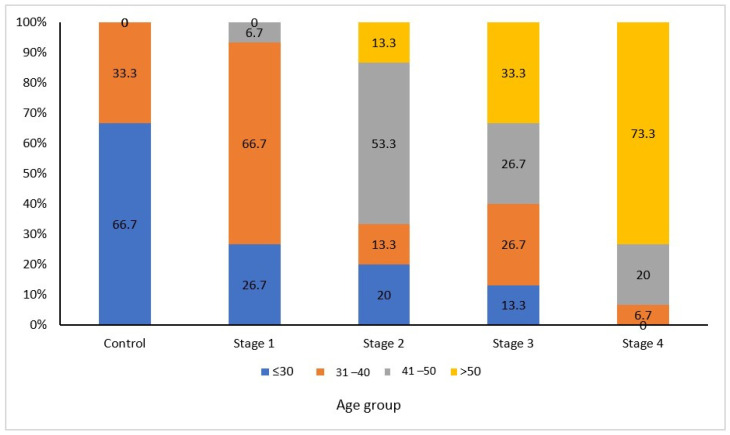
Age distribution among different stages of periodontitis.

Seventy-five individuals consisting of 31 females and 44 males aged 25 to 60 years were included in the study. Figure 2 presents the age distribution among the different stages.

Among the control group, the majority of the subjects (66.7%) were aged less than or equal to 30 years. In stage I, the majority were aged 31–40 years (66.7%). Among the Stage II cases, the majority were in the 41–50 years age group (53.3%), and in Stage IV, the majority were in the age group of over 50 years old (73.3%). This indicated the association of higher stage with higher age group and was found significant (*p* < 0.0001).

The mean ± standard deviation (SD) of % bone loss at stage I was 12.1 ± 2.6 with a median of 12.9 (10.0–14.7). The mean and median values increased with the advancement of stages from I to IV (Table 3). At stage IV, the mean ± SD was 77.0 ± 10.3, with a median of 77.6 (70.5–87.2). It clearly shows that the mean % bone loss increased with an increase in the stages and the difference was statistically significant (*p* < 0.0001).

Table 4 presents the comparison of the fractal dimension values among stages. In the control group, the mean ± SD of the fractal dimension value was 1.21 ± 0.07 with a median of 1.23 (1.19–1.27), gradually decreasing to 1.02 ± 0.11, 1.03 (1.01–1.08) in stage IV. It was found that the mean value of the fractal dimension decreased significantly with an increase in the stage (*p* < 0.0001).

The relationship between % bone loss and fractal dimension at different stages is presented in Table 5. The overall relationship between % bone loss and fractal dimension showed a significantly strong negative correlation of −0.739 (*p* < 0.0001). This indicated that an increase in % bone loss results in a decrease in FD value or vice versa. At stages I and II, % bone loss depicted a significant moderate negative correlation of −0.639 (*p* = 0.010) and −0.561 (*p* = 0.030) with FD, respectively. At stage III and stage IV, the correlation between % bone loss and the FD value was insignificant, with *p* = 0.418 and *p* = 0.196, respectively.

An ROC analysis was performed for four situations to determine the cut-off value of FD as a marker of periodontitis patients (Table 6).

**Figure 3:**
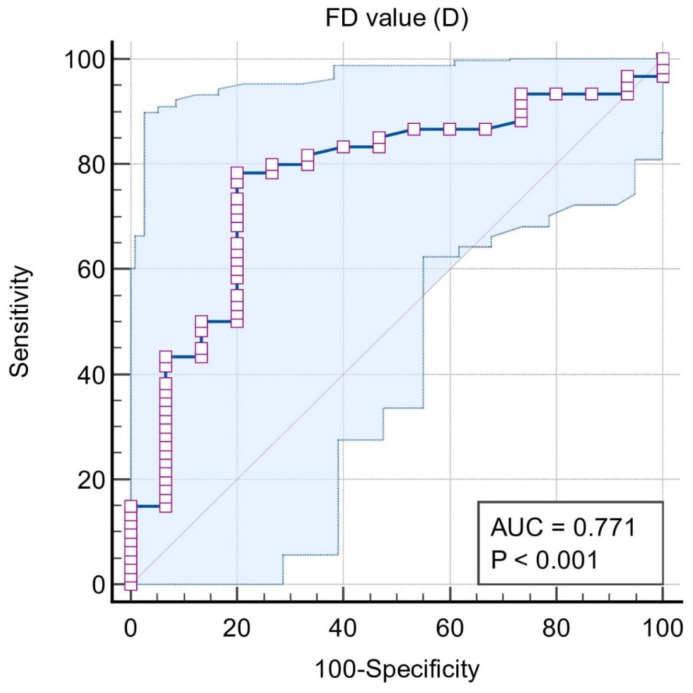
ROC curve for Situation #1.

Situation #1 is where the classification variable is stage I, II, III, and IV periodontitis = 1 and control is taken as 0. The cut-off FD was ≤1.188, sensitivity (95% CI) was 78.3 (65.8–87.9), and specificity (95% CI) was 80 (51.9–95.7). The area under the curve was 0.771 and was significant (*p* < 0.0001). According to situation #1, the cut-off value of FD was ≤1.188 to mark the periodontitis cases; however, the sensitivity was nearly 80% (Figure 3).

**Figure 4:**
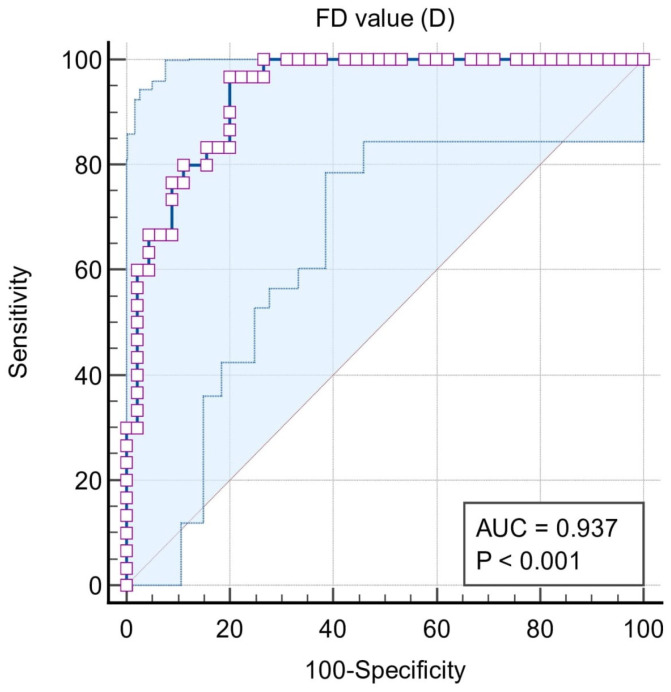
ROC curve for Situation #2.

Situation #2 is where stages III and IV periodontitis = 1, and control stage I and II = 0. The area under the curve was extremely high (0.937) and the cut-off value of the marker was ≤1.158. The sensitivity and specificity were 96.7% and 80%, respectively (Figure 4).

**Figure 5:**
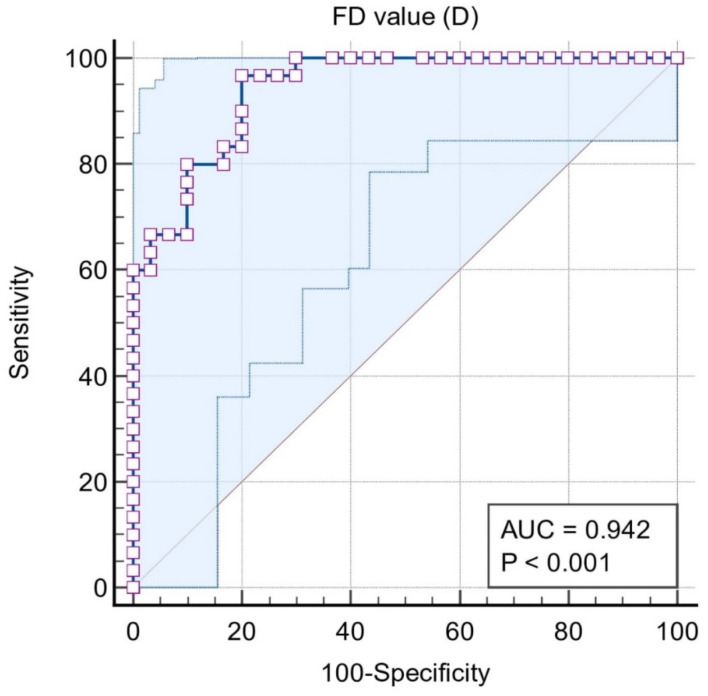
ROC curve for Situation #3.

In situation #3, the classification variables were stages III and IV = 1 and stages I and II = 0. In this case, the cut-off value of FD was also precisely the same as in situation #2. The sensitivity and specificity were also equally high as in situation #2. This indicated that a cut-off value of FD ≤ 1.158 is quite efficient for marking stage III and IV periodontitis (Figure 5).

**Figure 6:**
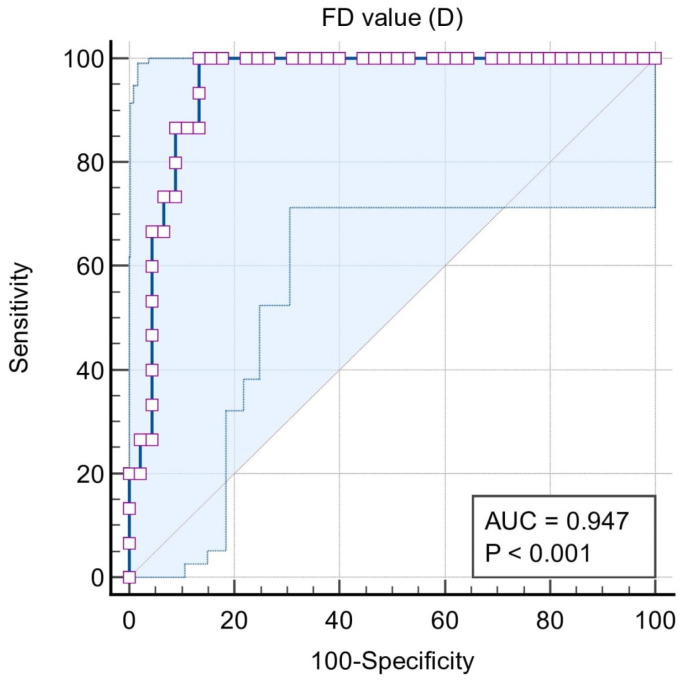
ROC curve for Situation #4.

In situation #4, the classification variables were stage IV = 1 and stages I, II, and III = 0. The area under the curve was extremely high (0.947), and the cut-off value of the marker was ≤1.102. The sensitivity (95% CI) and specificity (95% CI) was 100 (78.2–100) and 86.7 (73.2–94.9), respectively. The area under the curve was significant (*p* < 0.0001). According to situation #4, the true positive (sensitivity) rate was very high, i.e., 100%, compared to situations I, II, and III. FD ≤ 1.102 can be an efficient marker of stage IV periodontitis (Figure 6).

In another situation, situation #5, the classification variables were stages I and II = 1 and control = 0, which were taken to differentiate between periodontal health and early stages of periodontitis. The true positive rate was low, i.e., 56.67 (37.4–74.5), and the result was not found to be significant.

**Figure 7:**
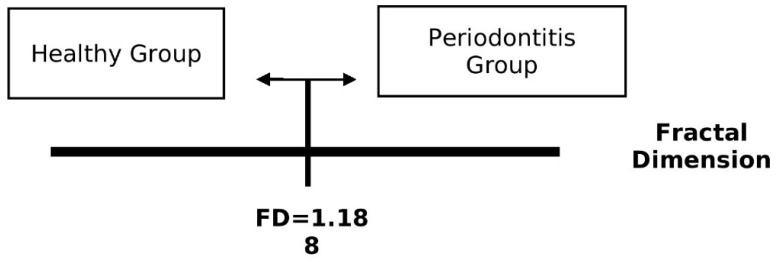
FD value to assign subjects into two periodontal groups: healthy or periodontitis.

From the ROC analysis, it can be outlined that patients were described as healthy if they had an FD above 1.188 and as having periodontal disease if they had an FD below 1.188, as shown in Figure 7.

**Figure 8:**
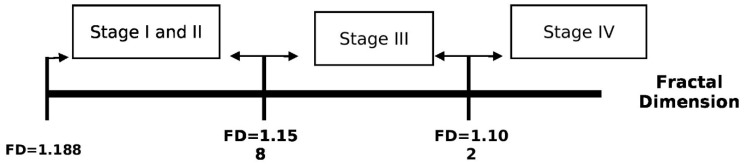
FD value as a marker to assign subjects to various stages of periodontitis.

In the periodontitis group, when the FD value is ≤1.188 but >1.158, it can be described as stage I and stage II periodontitis. When the FD value is ≤1.158, it can be attributed to stage III periodontitis; for the disease to be considered stage IV periodontitis, the FD value of ≤1.102 can be an efficient marker, delineating a severe stage of bone loss, as shown in Figure 8.

## 4. Discussion

Objective description of complex structures, which is impossible to perform with conventional methods, can be performed using fractal analysis, a non-invasive method. For the past several years, fractal analysis has been used as a quantitative method to evaluate the elementary components of complex biological structures in health and disease, including periodontal health. The software and techniques used for FD analysis are easy to master; however, it’s time-consuming. Even if the measuring algorithms differ, they follow the same basis summarized by the three steps: (1) measure the quantities of the object using various step sizes, (2) plot log (measured quantities) versus log (step sizes) and fit a least-squares regression line through the data points, and (3) estimate FD as the slope of the regression line.

FD is a mathematical tool amongst most other quantification methods that helps the clinician to perceive the quality of bone tissue. Apart from these high-standard images, high quality, as well as high precision image modality, is required. It is independent of radiation geometry and is a subtractive technique.

Previous studies have reviewed and analyzed the efficacy of FD for the differentiation of periodontitis and gingivitis and also for mild, moderate, and severe periodontitis according to the former classification of periodontitis, i.e., AAP classification, 1999 [18]. However, no studies were conducted to quantify the cancellous bone changes in different clinical presentations and the severity of the periodontal disease, according to the latest classification given by the World Workshop for Periodontal and Peri-Implant Diseases and Conditions, 2017.

The results of the present study showed that FD could be successfully used for the differentiation between healthy periodontium and periodontitis, as well as the different stages of periodontitis, using digital non-standardized clinical images. The method is independent of projection variations as proved previously, which makes it easier to be utilized in clinical evaluations [25,26].

In the last few decades, several efforts have been directed at detecting cancellous pattern changes on dental radiographs. However, earlier, the radiographic films were digitized using a scanner which caused a reduction in the grey values and information loss [27,28]. Such limitations were omitted in this study that accurately distinguished the healthy and the diseased groups. It has been advocated that when cone-beam CT images and micro-CT are used to calculate the FD, a more significant value of bone micro-architecture is found [29]. However, CBCT scans are not routinely used in general dentistry for diagnostics of periodontal diseases, while conventional dental radiography is safe, cheap, and widely available at nearly each dental office.

This study found a significant and unambiguous indication of the association of a higher stage of periodontitis with a higher age group. However, the variance in FD for age was of minor percentage. The differences found between healthy and periodontal groups in our study could not be attributed to the differences in age alone. Future research could be directed towards analyzing FD of healthy groups of different ages, to find if advanced stage of periodontitis and age are correlated.

Shrout et al. [25] used a caliper fractal analysis method to compare the cancellous pattern differences among healthy and moderate periodontal patients. It is known that there are many fractal calculation techniques, and different types of fractal methods produce different FD values. One of the most common techniques, the box-counting method provided by ImageJ to detect pattern changes induced by periodontitis, was used in the study. It was found that the mean ± SD of the fractal dimension value decreased significantly with an increase in the advancement of periodontitis. The periodontal disease was negatively correlated to FD; as periodontal disease increased, the fractal value decreased. This could be attributed to less complexity and space filling by cancellous arrangement with increasing demineralization of bone. In the present study, the relationship between FD and periodontal condition is similar to that found in Shrout’s caliper method of fractal analysis [25,30]. It is also in line with similar studies by Updike and Nowzari [18] and by Belgin and Serindere [31].

The overall relationship between % bone loss and fractal dimension showed a significantly strong negative correlation, except for stages III and IV. The reasons may be that in stages III and IV, both bone loss and FD value have attained their level of saturation that is negligible trabeculae were present approximating the FD values near to zero; as a result, the correlation is not discernible. With this finding, an ROC was performed to find a cut-off value of FD as a marker of stages III and IV for periodontitis. When the FD value is ≤1.188, it can be diagnosed as a periodontitis case. A cut-off value of FD ≤ 1.158 is quite efficient for marking stage III and IV periodontitis. To consider stage IV periodontitis, the FD value of ≤1.102 can be an efficient marker, delineating a severe stage of bone loss. Therefore, from the results, it can be portrayed that to differentiate a case from a healthy state to a state of periodontitis, the FD value has to be ≤1.188. Whereas, an FD value ≤ 1.158 marks stage III of periodontitis, and an FD value ≤ 1.102 outlines stage IV periodontitis. It was also analyzed if an FD measurement could differentiate between periodontal health and early stages of periodontitis. The results came out to be non-significant. However, this might be because the study was conducted in a small demographic. An ROC curve helps in illustrating the diagnostic ability of a tool. This study showed that fractal analysis is a highly sensitive tool and can detect minimal bony changes, hence it can be used as an efficient tool to diagnose periodontal disease. In a similar study by Updike and Nowzari [18], subjects were classified into a healthy group, a moderate group, and a severe periodontitis group. The ROC results for fractal analysis as a diagnostic tool showed a fair-to-good result to differentiate between healthy subjects and subjects with periodontitis. However, it was a poor tool for detecting differences between moderate and severe periodontal conditions [18]. This might be because the earlier classification (AAP 1999) did not provide a framework to demarcate the severity and complexity of the disease. Hence, the cut-off FD values determined in the results of this study can be used as an additional criterion in the staging of periodontitis according to the Classification of Periodontal and Peri-Implant Diseases and Conditions (WWDC), 2017.

Fractal dimension can be used in the future to study the bone and soft tissue gain after graft placement, as well as to study the implant surface characteristics and periimplantitis [32,33].

There are a few limitations to this study. The study identified alterations of cancellous bone using FD on digital images, but no comparison was made with other diagnostic methods, such as computed tomography, Feret diameter analysis, etc. The effect of the ROI location on the FD calculation was not proved, although it was demonstrated by Shrout et al. [25] that final ROI placement might not be necessary. However, there is a need for a consensus on the issue of ROI. Secondly, a study at two different time points is complex with the present FD protocols for the same individuals, since it is hard to select the same ROI in two images of the same anatomical region. This might be nullified by using standardized techniques of image registration. Hence, the future direction of the research demands implementation of a standardized protocol for ROI selection.

Additionally, some recently introduced compounds have been demonstrated to have a significant influence on the oral environment. Probiotics [34], lysates [35], and postbiotics [36] can modify clinical and microbiological parameters in periodontal patients, so these products should also be considered, in addition to age and other clinical parameters, in future trials to evaluate their long-term effects.

Moreover, two observers cannot select the ROI at the exact location, and intraobserver reliability could have been checked. In this study, White et al. [37] followed the protocol for image processing, where the grayscale images were converted into binary, based on a threshold value provided by the program. Alternate thresholding techniques may provide more consistent results. Future research can align towards developing less time-consuming software for the easy and practical use of FD analysis in day-to-day clinical practice. Also, in order to help future researchers, the technique needs to be corroborated on a greater number of subjects.

## 5. Conclusions

The present study showed a negative correlation of FD with an increasing stage of periodontitis. The lower values of FD with a higher stage may be attributed to the fact that, in the subjects with a higher stage of periodontitis, there is an increased spatial separation between the trabeculae. This eventually leads to less interconnection between trabeculae, loss of branching, and more presence of rounded trabeculae. Gender did not have any significant relation with FD, but for age, there was an inverse relationship. The FD value was calculated as a marker to distinguish each stage of periodontitis.

A few limitations were found and discussed. However, within the study’s limitations, it can be concluded that FD can be used as a quantitative and objective method for detecting cancellous bone in different stages of periodontal destruction. Image analysis of radiographs provides a non-invasive method for the clinician or researcher to extract data from pre-existing resources and at the same time can be used as a screening aid for the onset of cancellous pattern changes. It can be concluded that, to aid in our current clinical diagnosis, the box-counting method of fractal analysis could be used.

## Figures and Tables

**Table 1:** Inclusion and exclusion criteria for the study participants.

Inclusion Criteria	Exclusion Criteria
Patients within the age group of 30–60 years	Radiographs with poor resolution or diagnostic quality
Systemically healthy patients	Patients having diseases which may affect the bone density
Patients consenting to radiographic exposure	Patients not willing to have radiation exposure.
Patients having at least 20 remaining teeth	Teeth with dental caries extending in the cervical area.
Confirmed diagnosis of periodontitis (World Workshop Classification for Periodontal and Peri-Implant Diseases and Conditions, 2017)	Teeth with clinical attachment loss on the distal aspect of a second molar and associated with malposition or extraction of a third molar
	Teeth having previous root canal therapy or periapical lesions

**Table 2:** Study groups.

Study Group	Group Description
Group A	Healthy controls
Group B	Interdental CAL at the site of greatest loss 1–2 mm (STAGE I)
Group C	Interdental CAL at the site of greatest loss 3–4 mm (STAGE II)
Group D	Interdental CAL at the site of greatest loss ≥ 5 mm (STAGE III)
Group E	Interdental CAL at the site of greatest loss ≥ 5 mm (STAGE IV) Tooth loss due to periodontitis of ≥5 teeth.

**Table 3:** Bone loss [%] in different stages of periodontitis.

Stage	N	% Bone Loss					Kruskal–Wallis Test ‘*p*’ Value
		Mean	SD	Median	Range	Mean Rank	
Stage I	15	12.1	2.6	12.9 (10.0–14.7)	(7.0–14.9)	8.0	*p* < 0.0001
Stage II	15	25.4	4.7	25.3 (20.4–29.9)	(17.5–32.6)	23.0	
Stage III	15	57.9	6.1	58.8 (52.3–63.0)	(46.2–67.3)	38.8	
Stage IV	15	77.0	10.3	77.6 (70.5–87.2)	(54.7–91.5)	52.2	
Total	60	43.1	26.7	39.4 (15.6–64.9)	(7.0–91.5)		

SD = standard deviation.

**Table 4:** Comparison of fractal dimension values among stages of periodontitis.

Stage	N	FD Value					Kruskal–Wallis Test ‘*p*’ Value
		Mean	SD	Median	Range	Mean Rank	
Control	15	1.21	0.07	1.23 (1.19–1.27)	(1.03–1.29)	54.23	*p* < 0.0001
Stage I	15	1.21	0.06	1.21 (1.18–1.27)	(1.11–1.29)	53.00	
Stage II	15	1.19	0.05	1.18 (1.15–1.21)	(1.10–1.30)	46.10	
Stage III	15	1.11	0.05	1.12 (1.08–1.16)	(1.01–1.17)	25.73	
Stage IV	15	1.02	0.11	1.03 (1.01–1.08)	(0.63–1.10)	10.93	
Total	75	1.15	0.10	1.16 (1.09–1.22)	(0.63–1.30)		

SD = standard deviation.

**Table 5:** The relationship between % bone loss and fractal dimension at different stages of periodontitis.

Stages	% Bone Loss	FD Value (D)
Overall	Pearson Correlation	−0.739 **
	Sig. (2-tailed)	0.000
	N	60
Stage I	Pearson Correlation	−0.639 *
	Sig. (2-tailed)	0.010
	N	15
Stage II	Pearson Correlation	−0.561 *
	Sig. (2-tailed)	0.030
	N	15
Stage III	Pearson Correlation	0.226
	Sig. (2-tailed)	0.418
	N	15
Stage IV	Pearson Correlation	−0.353
	Sig. (2-tailed)	0.196
	N	15

* Correlation is significant at the 0.05 level (2-tailed). ** Correlation is significant at the 0.01 level (2-tailed).

**Table 6:** ROC analysis.

ROC	Classification Variables				
	Situation #1 (Stages I, II, III, and IV = 1, Control = 0)	Situation #2 (Stages III and IV = 1, Control, Stages I and II = 0)	Situation #3 (Stages III and IV = 1, Stages I and II = 0)	Situation #4 (Stage IV = 1, Stages I, II, and III = 0)	Situation #5 (Stage I and II = 1, Control = 0)
Sample size	75	75	60	60	45
Area under the ROC curve (AUC)	0.771	0.937	0.942	0.947	0.614
95% Confidence interval b	0.659 to 0.860	0.856 to 0.980	0.850 to 0.986	0.856 to 0.988	0.458 to 0.756
Significance level P (Area = 0.5)	0.0001	<0.0001	<0.0001	<0.0001	0.2228
Associated criterion	≤1.188	≤1.158	≤1.158	≤1.102	≤1.188
Sensitivity (95% CI)	78.3 (65.8–87.9)	96.7 (82.8–99.9)	96.7 (82.8–99.9)	100 (78.2–100)	56.67 (37.4–74.5)
Specificity (95% CI)	80 (51.9–95.7)	80 (65.4–90.4)	80 (61.4–92.3)	86.7 (73.2–94.9)	80.00 (51.9–95.7)

## Data Availability

The data presented in this study are available on request from the corresponding author.
